# Efficient adeno-associated virus serotype 5 capture with affinity functionalized nanofiber adsorbents

**DOI:** 10.3389/fbioe.2023.1183974

**Published:** 2023-05-16

**Authors:** Salomé Neto, João P. Mendes, Susana B. Dos Santos, Anita Solbrand, Manuel J. T. Carrondo, Cristina Peixoto, Ricardo J. S. Silva

**Affiliations:** ^1^ iBET, Instituto de Biologia Experimental e Tecnológica, Oeiras, Portugal; ^2^ ITQB NOVA, Instituto de Tecnologia Química e Biológica António Xavier, Universidade Nova de Lisboa, Oeiras, Portugal; ^3^ Cytiva, Cambridge, United Kingdom; ^4^ Cytiva, Uppsala, Sweden

**Keywords:** AAV5, affinity chromatography, gene therapy, non-woven materials, cellulose-based nanofibers

## Abstract

Adeno-associated viruses (AAVs) are one of the most promising tools for gene therapy applications. These vectors are purified using affinity and ion exchange chromatography, typically using packed beds of resin adsorbents. This leads to diffusion and pressure drop limitations that affect process productivity. Due to their high surface area and porosity, electrospun nanofiber adsorbents offer mass transfer and flow rate advantages over conventional chromatographic media. The present work investigated the use of affinity cellulose-based nanofiber adsorbents for adeno-associated virus serotype 5 (AAV5) capture, evaluating dynamic binding capacity, pressure drop, and AAV5 recovery at residence times (RT) less than 5 s. The dynamic binding capacity was found to be residence time-dependent, but nevertheless higher than 1.0 × 10^14^ TP mL^−1^ (RT = 1.6 s), with a pressure drop variation of 0.14 MPa obtained after loading more than 2,000 column volumes of clarified AAV5 feedstock. The single affinity chromatography purification step using these new affinity adsorbents resulted in 80% virus recovery, with the removal of impurities comparable to that of bead-based affinity adsorbents. The high binding capacity, virus recovery and reduced pressure drop observed at residence times in the sub-minute range can potentially eliminate the need for prior concentration steps, thereby reducing the overall number of unit operations, process time and costs.

## 1 Introduction

Adeno-associated viruses are emerging as one of the leading platforms for the treatment of a wide range of medical conditions. The approval of five gene therapy products, including Glybera^®^ (AAV1), Luxturna^®^ (AAV2), Zolgensma^®^ (AAV9), Roctavian^®^ (AAV5) and Hemgenix^®^ (AAV5), illustrate the great potential of these vectors (Duan, 2018; Pasi et al., 2020; Roland et al., 2023). Given these recent success stories, the demand for high vector quantities and reduced timelines highlight the need for more efficient production technologies (Penaud-Budloo et al., 2018).

During research and development, recombinant AAVs are typically purified, after expression in mammalian or insect cells, using density gradient ultracentrifugation methods (Carvalho et al., 2021; Moleirinho et al., 2019). Although these methods can be applied regardless of the serotype, resulting in highly pure AAVs preparations with two or three rounds of ultracentrifugation, several disadvantages have been reported. Product yield is low, significant time-consuming manual labor is required, and scale-up is challenging (Qu et al., 2015; Adams et al., 2020). In addition, some of the compounds used to form the gradient are cytotoxic (e.g., CsCl) and therefore require additional buffer exchange steps (Qu et al., 2015; Adams et al., 2020). Alternatively, aqueous two-phase systems have been demonstrated as a potential and economical method to purify enveloped and non-enveloped viruses (Turpeinen et al., 2021). The method consists of mixing a water-soluble polymer and a salt, above a critical concentration that results in two immiscible phases. Guo et al. (2012), Kimura et al. (2019) and Yu et al. (2020) report a two- and three-phase partitioning of PEG and/or ammonium sulfate combined with one or two rounds of iodixanol or CsCl gradient ultracentrifugation to obtain high purity levels of AAVs (>90%) (Guo et al., 2012; Kimura et al., 2019; Yu et al., 2020).

The use of chromatography and filtration approaches is generally preferred as these can be more readily scaled to become compatible with industrial production scales (Carvalho et al., 2021; Moleirinho et al., 2019). Chromatography modes using ion exchange, hydrophobic interaction and affinity are commonly used for virus purification (Kaludov et al., 2002; Qu et al., 2015; Tomono et al., 2018; Moleirinho et al., 2019; Adams et al., 2020), the latter allowing to achieve high levels of purity while reducing the number of purification steps (Qu et al., 2015). Due to the selective capacity of affinity chromatography and the development of specific affinity ligands for different AAVs serotypes, several AAVs purification studies report the use of an affinity capture step followed by an ion exchange polishing used to deplete empty particles (Nass et al., 2018; Srivastava et al., 2021; Singh and Heldt, 2022).

Affinity chromatography media for AAVs purification are typically found in bead format, with reported binding capacities between 1 × 10^13^ and 2 × 10^14^ viral particles per mL of packed bed (Fisher Scientific, 2017; Cytiva, 2018; Cytiva, 2020a; Repligen Corporation, 2022). These media are already available in a prepacked format eliminating the need for packing, cleaning, and validation. Nevertheless, the binding capacity in these adsorbents is highly dependent on the flow rate due to the slow intraparticle diffusion, with higher flow rates leading to higher-pressure drops, and reduced binding capacity. This results in lower productivity, poor ligand usage and longer process times (Orr et al., 2013). Tangential flow filtration is usually performed to reduce the loading volume for the chromatography process, and subsequently improve process time (Moleirinho et al., 2019). However, the use of convective stationary phases, such as membranes and monoliths, overcomes the mass transfer limitations of bead-based materials, allowing higher flow rates at lower operating pressures, reducing processing time and the need for a previous concentration step. (Lalli et al., 2020; Lemma et al., 2021).

Most adsorptive membrane materials are based on regenerated cellulose, polyethersulfone, and polyvinylidene fluoride. The use of electrospun cellulose-based nanofibers forming porous matrices with an open and well-defined structure without dead-end pores has been demonstrated for antibody purification (Boi, 2007). The electrospinning process involves passing a dissolver polymer (viscous solution) by a charged microneedle at high voltage to form a non-woven fiber matrix. Due to the low solubility of cellulose in common solvents, cellulose acetate is used and then regenerated to cellulose via hydroxide treatment. These nanofiber adsorbents combine high surface area and porosity, allowing high flow rates and convective mass transfer advantages (Hardick et al., 2013; Dods et al., 2015). This technology is the base of HiTrap Fibro™ Prism A protein A developed by Cytiva, for the capture of monoclonal antibodies (mAbs) and Fc-containing recombinant proteins (Cytiva, 2020b). Reports show that the observed binding capacities for the Fibro™ PrismA units are comparable to the ones obtained with bead chromatography, with 30-fold higher productivity and a significant reduction of 160 times in the residence time required for HiTrap™ PrismA column (Davis et al., 2021).

This manuscript describes the affinity purification of AAV5 from clarified feedstock materials using cellulose-based nanofibers adsorbents. Firstly, breakthrough curves were generated through a series of frontal experiments. These were fitted to estimate the saturation capacity and determine dynamic binding capacity at 10% of breakthrough. Afterwards, the elution recovery and its dependence on residence time and loading volume were assessed. The robustness and reproducibility of the separation were then analysed for a fixed-volume injection of clarified material below the dynamic capacity determined. Additionally, due to the high flow rates possible, the pressure profile of these units was also analysed for water-like solutions and compared with clarified AAV5 materials.

## 2 Materials and methods

### 2.1 AAVs production

Human Embryonic Kidney cells 293T (HEK 293T) ACS-4500, adapted to suspension, were purchased from ATCC (Virginia, United States). These were routinely sub-cultured to 0.6 × 10^6^ cells mL^−1^ every 48 h when cell concentration reached 2–3 × 10^6^ cells mL^−1^ using vented non-baffled shake flasks with BalanCD HEK293 medium (Irvine Scientific, California, United States) supplemented with 4 mM of GlutaMAX (Gibco, Fisher scientific, Hampton, United States) under a humidified atmosphere of 5% CO_2_ in air at 37°C with controlled agitation (orbital diameter of 25 mm, 90 rpm). The AAVs feedstock generated for this study was obtained in a 20 L Biostat^®^ D-DCU (Sartorius, Göttingen, Germany) stirred-tank bioreactor (STB) equipped with two Rushton impellers and a ring-sparger for gas supply. The pO_2_ was set to 40% of air saturation and was maintained by varying the agitation rate (70–200 rpm), the percentage of O_2_ in the gas mixture (0%–100%), and the gas flow rate (0.01–0.04 vvm). The pH value was maintained by the automatic addition of either 1 M of NaHCO_3_ or CO_2_ within the gas mix. Cells were transfected with a DNA plasmid solution containing 1.5 µg of total plasmid DNA per 10^6^ cells. This mix included pDP 5 (reference: PF0435) and p-AAV-ssGFP (reference: PF1451), at a molar ratio of 1:1 (PlasmidFactory, Bielefeld, Germany), diluted in a specific volume of supplemented culture medium, corresponding to 5% of culture volume. Additionally, PEI MAX (PolySciences, Bergstrasse, Germany) transfection reagent was added with a 1:2 µg DNA/µg PEI ratio between total plasmid and reagent. This solution was incubated at room temperature for up to 15 min before addition.

### 2.2 Cell lysis and clarification

Cells were lysed 72 h after transfection with 50 mM Tris-HCl, 1.0 (v/v) % Tween 20, and 2 mM of MgCl_2_, at pH 8, followed by the addition of 50 Units per mL of Benzonase (catalog number: 1.01656.0001, Merck Millipore, Darmstadt, Germany). To prevent aggregation, salt-concentrated solutions of MgSO_4_ and NaCl were supplemented to a final concentration of 37.5 mM and 200 mM, respectively. The cell lysate was harvested and clarified with two different filter trains. The first consisted of a depth filter capsule with 0.2 m^2^ of the effective filtration area and retention range between 6 and 30 μm (Supracap™ 100, Pall Corporation, New York, United States) followed by a second filtration stage with a Gamma–Irradiatable MidiCap filter of 0.45 m^2^ surface area and 0.2 μm of pore size (Sartopore^®^ 2, Sartorius Stedim, Göttingen, Germany) operated at flow rate of 600 mL min^−1^.

### 2.3 Chromatography experiments

The chromatography experiments were performed at room temperature of 20°C using an ÄKTA avant 25 (Cytiva, Uppsala, Sweden). A device of 0.4 mL of volume, consisting of a cellulose-based fiber matrix with geometry and flow properties similar to the commercially available HiTrap Fibro™ PrismA was provided by Cytiva (Uppsala, Sweden) (Hardick et al., 2013; Dods et al., 2015; Cytiva, 2020b). The fiber matrix is functionalized with an affinity ligand designed for purifying AAVs serotypes, including 1, 2, 3, and 5, similar to the commercially available affinity chromatography resins Capto AVB or Sepharose AVB (Cytiva, 2018; Cytiva, 2020a). The device was equilibrated with 15 column volumes (CV) of 20 mM Tris-HCl (Merck KGaA, Darmstadt, Germany), 500 mM NaCl (Merck KGaA, Darmstadt, Germany), and pH 8. After sample loading, the unit was washed with 30 CV of equilibrium buffer. The AAVs were eluted with 40 CV of 100 mM Glycine (Sigma-Aldrich, Missouri, United States), pH 2.5 and neutralized with 1 M Tris-HCl (Merck KGaA, Darmstadt, Germany), pH 9.

### 2.4 Breakthrough curve fitting and permeability estimation

The prediction of breakthrough curves and adsorber capacity is frequently performed recurring to Thomas’s model, which assumes negligible external and internal diffusion resistances, Langmuir kinetics of adsorption-desorption and second-order reversible reaction kinetics (Atar et al., 2011; Futalan et al., 2011; López-Cervantes et al., 2018; Patel, 2019).

The linearized form of the Thomas model is as follow:

$$\ln\left(\frac{C_{0}}{C_{t}}-1\right)=\frac{k_{Th}q_{0}w}{Q}-k_{Th}C_{0}t$$ (1)

Where C_0_ and C_t_ are the column inlet and outlet AAVs concentration at time t, k_Th_ is Thomas rate constant, q_0_ is the saturation capacity of the device, w is the mass of the adsorbent and Q is the flow rate. Eq. 1 was modified to take into consideration the volume of the adsorber, replacing w with the packed nanofiber volume (0.4 mL). To keep Eq. 1 dimensionally consistent, q_0_ should therefore be expressed in terms of adsorbed AAVs per unit volume of packed nanofiber adsorber.

Permeability of the nanofiber adsorber can be obtained by the linear fitting of the superficial velocity *versus* ∆ P/L (Herigstad et al., 2015; Lemma et al., 2021), according to Darcy’s Law:

$$v=\frac{k}{\mu }\frac{∆P}{L}$$ (2)

Where v is the superficial velocity, ∆ P is the pressure drop along the adsorber height (L), µ is the viscosity of the fluid, and k is the flow permeability of the porous medium.

### 2.5 Analytics

#### 2.5.1 Total protein and ds-DNA quantification

Total protein and ds-DNA content were assessed with specific assays according to the manufacturer’s instructions of each. The total protein content was quantified using a BCA Protein Assay Kit (Thermo Fisher Scientific, Rockford, United States) and total ds-DNA was quantified with a Quant-iT^™^ Picogreen^®^ dsDNA assay kit (P7589, Invitrogen^™^, Waltham, MA, United States). Bovine serum albumin (BSA) and λ-DNA (provided by the kits) were used for the calibration curves of total protein and ds-DNA, respectively. Samples were diluted 2 to 256-fold to avoid interferences with the method. Absorbance concerning protein quantification and fluorescence with ds-DNA quantification was measured with an Infinite 200 PRO NanoQuant (Tecan, Männedorf, Switzerland) microplate reader.

#### 2.5.2 Dynamic light scattering

Dynamic light scattering (DLS) was performed using a Zetasizer Nano ZS from Malvern (Worcestershire, United Kingdom) with Zetasizer software associated, to determine the size distribution of purified AAVs. It was performed 3 measurements for the sample. Each measurement consists of 10 runs with a duration of 10 s in disposable cuvettes, at 25°C. The samples were diluted at 1:40 in PBS (EMD Millipore, Burlington, United States) at pH 7.4 and filtered at 0.2 μm, to minimize the effect of particle-particle interactions.

#### 2.5.3 AAVs quantification

Total AAVs particle concentration (TP) was determined with a conformational AAV5 ELISA assay (Progen Biotechnik GMBH, Heidelberg, Germany) according to the manufacturer’s instructions. The absorbance was quantified at 450 nm on an Infinite 200 PRO NanoQuant (Tecan, Männedorf, Switzerland) microplate multimode reader using a clear 96-plate well provided in the kit. The samples were applied with three dilutions (in duplicate).

#### 2.5.4 HPLC-SEC

HPLC-SEC analysis was performed using a SRT SEC-1000 column (4.6 × 300 mm) (Sepax Technologies, Delaware, United States). The column was equilibrated with 10 column volumes of a mobile phase containing 150 mM phosphate buffer (Merck KGaA, Darmstadt, Germany), at pH 7, at 0.35 mL min^−1^. At the same flow rate, 5 µL of the sample previously diluted 1:4 in the mobile phase was loaded onto the column. The stationary and mobile phases were contained within an HPLC Vanquish system (ThermoFisher Scientifc, Waltham, United States) equipped with UV diode array UV and fluorescence detectors. Chromeleon software version 7.3 was used to control and analyse UV absorbance and fluorescence data. All steps post-injection were performed at 25°C.

#### 2.5.5 SDS-PAGE and western blot

The protein profile analysis was carried out in NuPage 4%–12% Bis-Tris Gel (Invitrogen) after protein denaturation. The gels were run for 45 min at a constant voltage of 200 V and stained with Coomassie Instant Blue (Expedeon Ltd., Cambridge, MA, United States) following membrane transfer. The iBlot system (Invitrogen, Thermo Fisher Scientific, Rockford, United States) was used to transfer the AAVs proteins to the PVDF membrane. The membrane was blocked in Tris-buffered saline with 0.1% (w/v) of Tween 20 with 5% (w/v) skim milk powder for microbiology (Merk Millipore, Darmstadt, Germany) for 1 h. Then, immunostaining was performed overnight using anti-AAV VP1, VP2, and VP3 mouse mono IgG1 clone B1 (Progen Biotechnik GMBH, Heidelberg, Germany) primary antibody. Afterwards, the membranes were washed and incubated with the secondary antibody, ECL anti-mouse HRP linked IgG NA931-100 μL (Cytiva, Uppsala, Sweden) for 1 h. The proteins were revealed using ECL Detection Reagent (Cytiva, Uppsala, Sweden) in a ChemiDoc XRS + System (Bio-Rad, California, United States).

#### 2.5.6 Transmission electron microscopy

Transmission electron microscopy (TEM) was performed to assess the presence and quality of AAVs. A volume of 5 µL of the sample was adsorbed onto a Formvar-coated 150 mesh copper grid from Veco (Science Services, Munich, Germany) for 10 min. The grid was washed with sterile water and a solution of 2% uranyl acetate in sterile water was added for 5 min and left to dry at room temperature. The image was taken with a Hitachi H-7650 120 kV electron microscope (Hitachi High-Technologies Corporation, Tokyo, Japan). For each sample 20 images were taken at different scales (500, 200 and 100 nm) from at least two different areas of the grid, that were representative of the entire sample.

## 3 Results and discussion

### 3.1 Pressure drop and adsorber permeability

**FIGURE 1:**
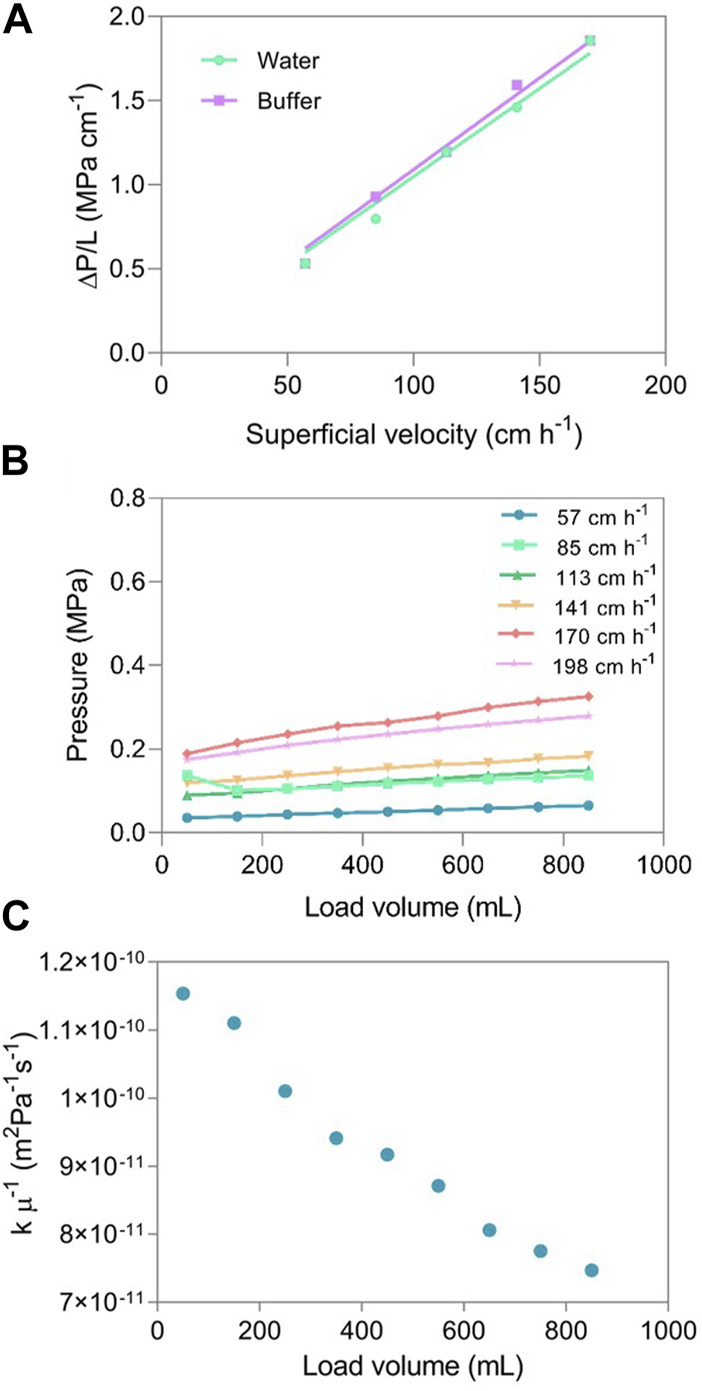
**(A)** Pressure drop normalized with membrane bed height for water (y = 0.0105x with R^2^ = 0.998) and for equilibration buffer (y = 0.0109x with R^2^ = 0.999) *versus* the superficial velocity in a unit with a volume of 0.4 mL and a diameter of 2.6 cm packed with two layers of membrane; **(B)** ΔP pressures of the nanofiber during the loading of more than 2100 CV of AAV5 clarified material at superficial velocities of 57, 85, 113, 141, 170 and 198 cm h^−1^ and **(C)** representation of k *µ* ^−1^ as a function of a load volume above 2100 CV of AAV5 clarified material.

Pressure drop in chromatography media limits the superficial velocity and consequently flow rate, affecting process productivity. The nanofiber adsorber permeability, using water and running buffer, were determined under a range of superficial velocities between 57 and 170 cm h^−1^ (corresponding to flow rates of 5.0–15.0 mL min^−1^). Additionally, pressure drop across the chromatographic support was also evaluated using clarified AAV5 material and compared against water and equilibration buffer. The linear relationship between pressure drop and flow rate, demonstrated in Supplementary Figure S1, indicates, not only, a laminar flow regime, but as well, the stability of the porous nanofiber matrix by not contracting at higher flow rates for water and equilibration buffer (Mihelič et al., 2005). Under these circumstances, the Darcy law is valid, and the permeability can be obtained through a linear fitting of the superficial velocity as a function of the pressure drop along the unit length. As the equilibrium buffer presents a low solid concentration, it was assumed that its viscosity is equal to the water, 10^–3^ Pa s (Ho and Zydney, 2000). Figure 1A shows the linear relation between ΔP/bed height (ΔP/L) *versus* the superficial velocity; the slope was used to calculate the flow permeability for water, 2.65 × 10^−15^ m^2^ and for buffer, 2.55 × 10^−15^ m^2^. The values obtained are in the same order of magnitude as the values obtained by Trilisky et al. for CIM DEAE disks (6.30 × 10^−15^ m^2^) (Trilisky et al., 2009), Herigstadt et al. for CIM protein A disks (5.74 × 10^−15^ m^2^) (Herigstad et al., 2015) and Lemma et al. for PBT membranes (2-3×10^−15^ m^2^) (Lemma et al., 2021).

The chromatographic purification of biological materials has to deal with colloids, such as lipids, cell debris, and carbohydrates, as well as soluble components, such as proteins and nucleic acids. Although cells and cell debris are usually removed during clarification, the remaining components can adversely affect adsorption performance, especially in cases of prolonged exposure, as in the case of a capture step. These components can occupy the interparticle pore space by competing for the binding groups or by blocking the pores of the chromatography medium. Consequently, this can be manifested as reduced resolution, reduced adsorption capacity, or increased pressure drop, all of which affect the efficiency of the purification step (Siu et al., 2006). The impact of the loading volume on pressure drop during the capture step was evaluated using AAV5 clarified material. The pressure drop was monitored at different superficial velocities (57–198 cm h^−1^) and is reported in Figure 1B as averages over 100 mL loading intervals. These were used to calculate the variation of the group k µ^−1^ of the Darcy equation over the loading of more than 2100 CV. As seen in Figure 1C, k µ^−1^ decreases over the continued loading. As the viscosity of the AAVs feedstock solution remains constant over the loading volume, this decrease can therefore be explained by a reduction in the permeability of the nanofiber adsorbents as a consequence of such high loading volumes. Considering that the amount of loaded material is approximately twice the highest value calculated for the dynamic binding capacity of the unit, as demonstrated in the next section, the observed variation in permeability value is lower than 40%.

### 3.2 Breakthrough curve fitting and dynamic binding capacity

**FIGURE 2:**
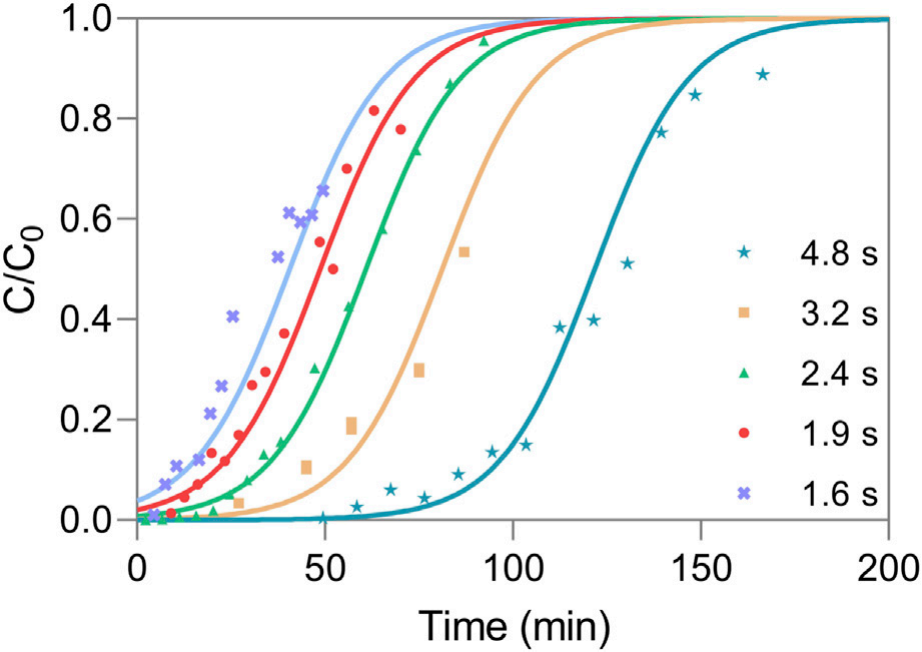
Fitting of breakthrough experiments for different residence times, at a feed concentration of 5.45 × 10^11^ TP mL^−1^ and with k_Th_ of 1.46 × 10^−13^ mL min^−1^ TP^−1^. The time points represent the experimental data and the lines represent the chromatography model fitting.

**FIGURE 3:**
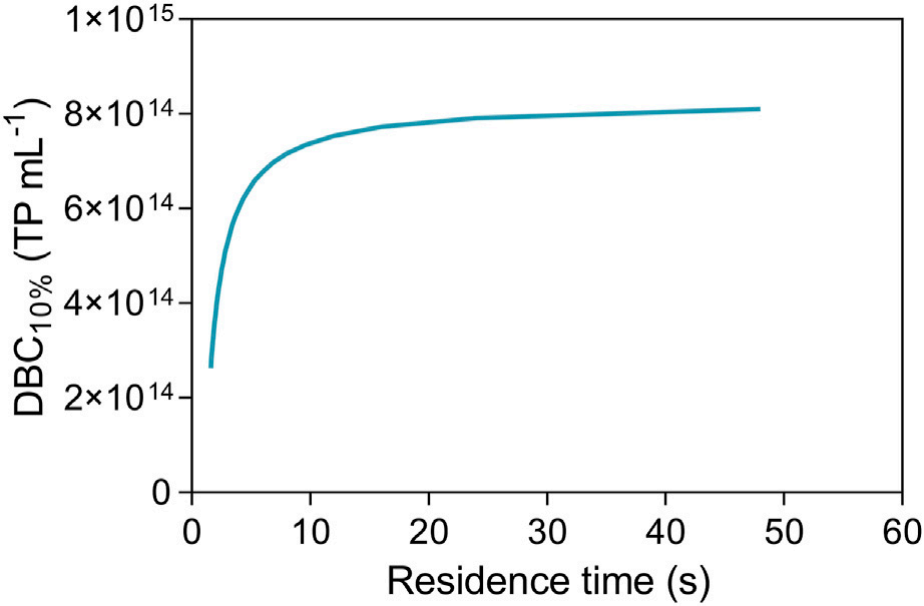
Dependence of the dynamic binding capacity at 10% with the residence time.

To assess the dynamic response, the shape and time of breakthrough curves were obtained from frontal experiments performed with AAV5 clarified samples at a feed concentration of 5.45 × 10^11^ TP mL^−1^. The collected samples during the unit loading were analyzed using ELISA. The breakthrough curves generated with experimental data were fitted simultaneously (Figure 2) using the Thomas model. This model is applied for adsorption processes where external and internal diffusion limitations are negligible (Futalan et al., 2011), and can be suitable for convective materials with some degree of approximation. In this sense, the saturation capacity and Thomas kinetic constant were assumed as independent of the residence time. The model fitting in the range of residence times scoped allowed to determine the saturation capacity of the nanofibers (q_0_) of 8.29 × 10^14^ TP mL^−1^. Additionally, the modelling of the breakthrough curves also allows the estimation of the unit’s dynamic binding capacity (DBC), which is used as a measure of the performance of the chromatographic material. The estimated DBC for a percentage of 10% of the breakthrough (DBC_10%_) at different residence times is reported in Figure 3, where for longer residence times the estimated DBC_10%_ approaches q_0_. This is corroborated in Figure 2, where the breakthrough curves move to earlier elution times as the residence time is decreased. The same effect in the breakthrough time would be observed if the inlet concentration is increased for a given flow rate, assuming that the nanofibers explored are dominated by convective flow, with presumably negligible resistance to mass transfer (Sridhar, 1996; van Beijeren et al., 2012).

### 3.3 Residence time and column overload effect on recovery

**FIGURE 4:**
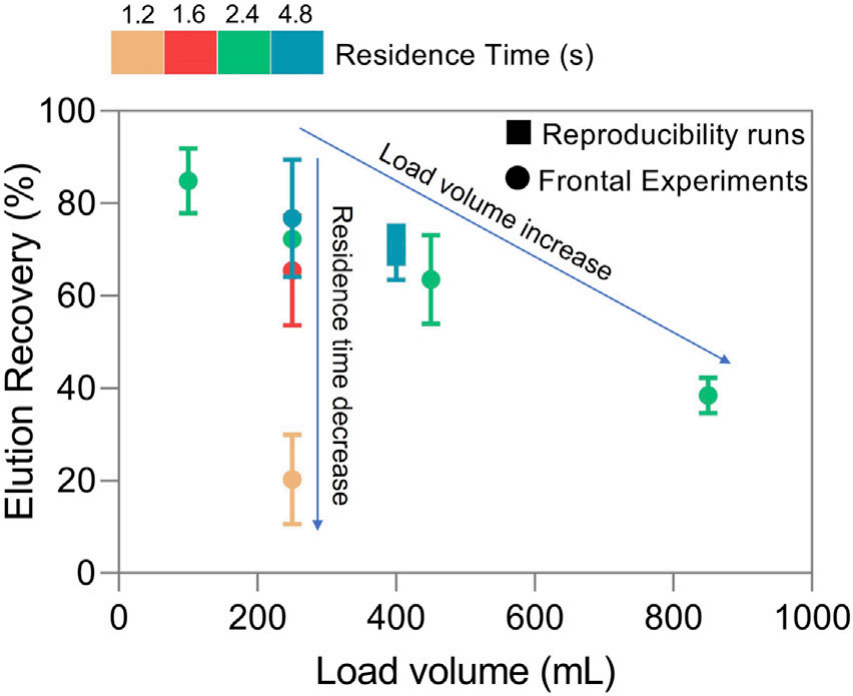
Elution recovery for different residence times and loading volumes.

The load volume effect in the AAVs elution recovery was tested using a set of chromatographic runs where the load volume was varied (100–850 mL or 250–2125 CV) for a fixed residence time (2.4 s). The results are reported in Figure 4. As observed, the elution recovery varies from 85% to approximately 40% with the load volume increase. This is in line with the data of Figure 3, where for a constant residence time, the continuous increase in the load volume leads to exceeding the DBC_10%_ at that point. As a result, this surplus is no longer adsorbed to the nanofiber and the final recovery decreases.

In the second stage, to analyze the effect of residence time in the elution recovery, this parameter was varied from 4.8 to 1.2 s using a fixed loading volume of 250 mL (625 CV) of feedstock (Figure 4). In this case, the AAVs recovery varies between 77% and 20%, following Figure 3, for a fixed volume, as the residence time decreases, the dynamic binding capacity of the nanofibers decreases, reducing virus adsorption and consequently recovery. Additionally, the content in total protein and ds-DNA of elution samples was analyzed (Table 1), revealing a removal of around 90% for total protein and between 80–90% for ds-DNA. As expected, results show that overloading the nanofibers or decreasing residence time is reflected in flowthrough losses as adsorptive capacity is exhausted. This can be minimized with applications such as continuous chromatography where media saturation is desired and loading zones composed of several interconnected adsorptive beds are used (Araújo et al., 2007; Silva et al., 2015; Mendes et al., 2021).

**TABLE 1:** Characterization of the affinity eluted samples from all process conditions of Figure 4 by total protein, ds-DNA and, respective reduction percentage from the clarified feedstock (total protein: 2.8 mg mL^-1^ and ds-DNA: 0.2 μg mL^-1^).

Samples		Total Protein		Total ds-DNA	
Residence time (s)	Load volume (mL)	Concentration in eluate (mg mL^-1^)	Reduction percentage (%)	Concentration in eluate (µg mL^-1^)	Reduction percentage (%)
1.2	250	0.15	95	0.02	90
1.6	250	0.19	93	0.03	85
2.4	100	0.14	95	0.02	90
	250	0.26	91	0.03	85
	450	0.22	92	0.02	90
	900	0.27	90	0.03	85
4.8	250	0.34	88	0.04	80
	400*	0.61 ± 0.01	78 ± 1	0.03 ± 0.01	84 ± 4

*Affinity eluates from reproducibility study (n=3).

### 3.4 Reproducibility

The reproducibility of the purification was evaluated at a residence time of 4.8 s, using new nanofiber adsorbents for each run (total of 3). The RT was selected based on Figure 3 where for RT below 4.8 s DBC would be greatly affected. Conversely, higher RT would not significantly improve DBC, as DBC tends to a constant value. All the procedures were automated, and the sample volume injected corresponds approximately to the calculated value of DBC_10%_. The chromatograms are depicted in Supplementary Figure S2.

The three chromatographic experiments show a similar UV absorption profile. The eluates were analysed by ELISA to determine AAVs recovery. The average value obtained, 71.3% is following the previous observations of the impact of residence time on loading volume (Figure 4). The sample loading of each run varied △P from 0.05 MPa (initial) to 0.08 MPa (end). These values are in accordance with what is observed in Figure 1C for the same flow rate. The quantification of flowthrough pools of each run was close to the lower detection limit of ELISA method used and highly prone to quantification errors. Considering the elution recoveries obtained and the possible low concentration of AAVs in flowthrough pools it is plausible that some of the AAVs adsorbed are only removed from the nanofibers during harsher elution conditions or cleaning in place.

**FIGURE 5:**
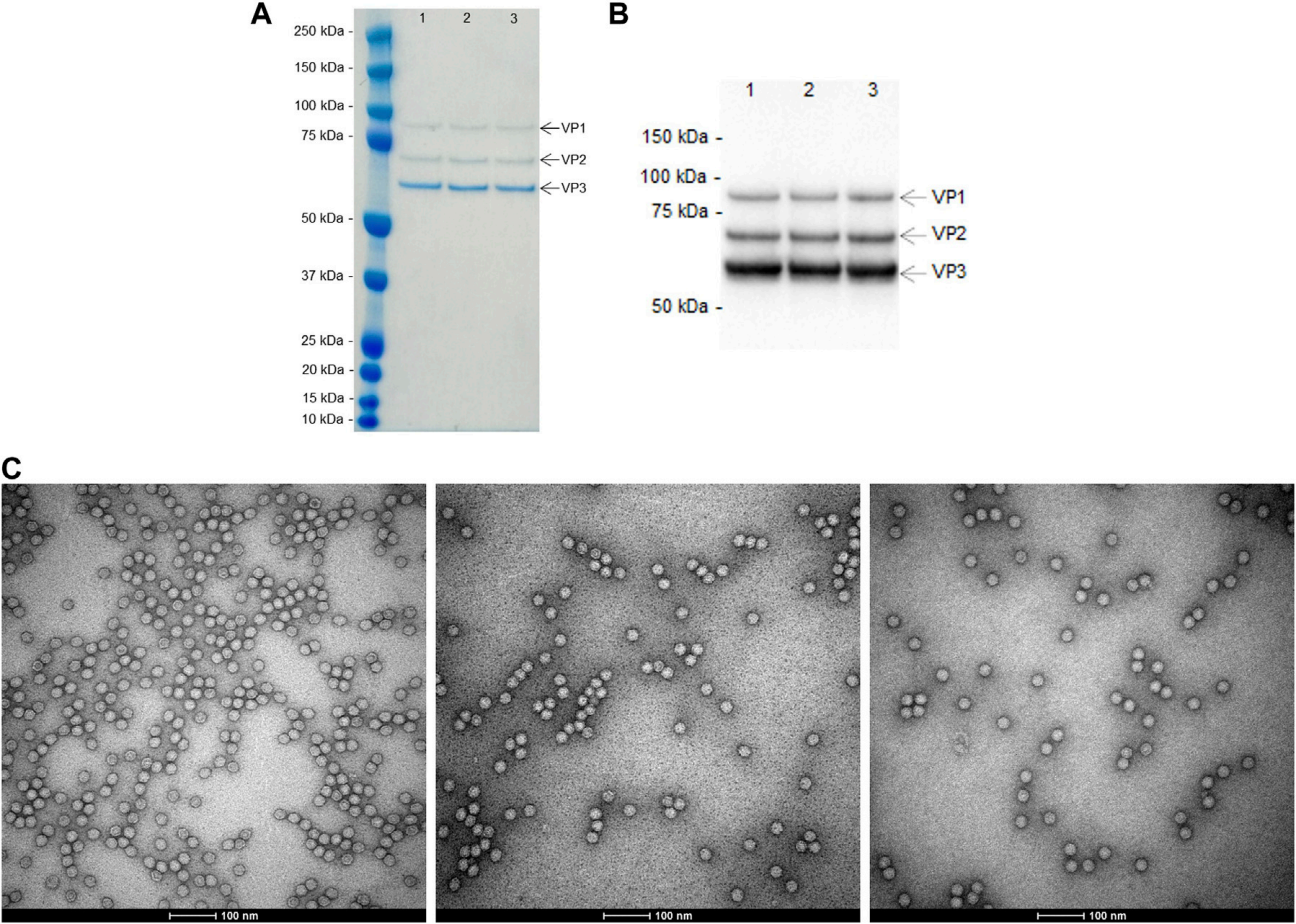
Characterization of the eluted peaks from the reproducibility study (residence time of 4.8 s and loading up to DBC_10%_) by: **(A)** SDS-PAGE Gel; **(B)** western blot targeting AAV capsid proteins (VP1, VP2, and VP3); and **(C)** transmission electron microscopy (representative images from run 1 - left, run 2 - middle and run 3 - right).

**FIGURE 6:**
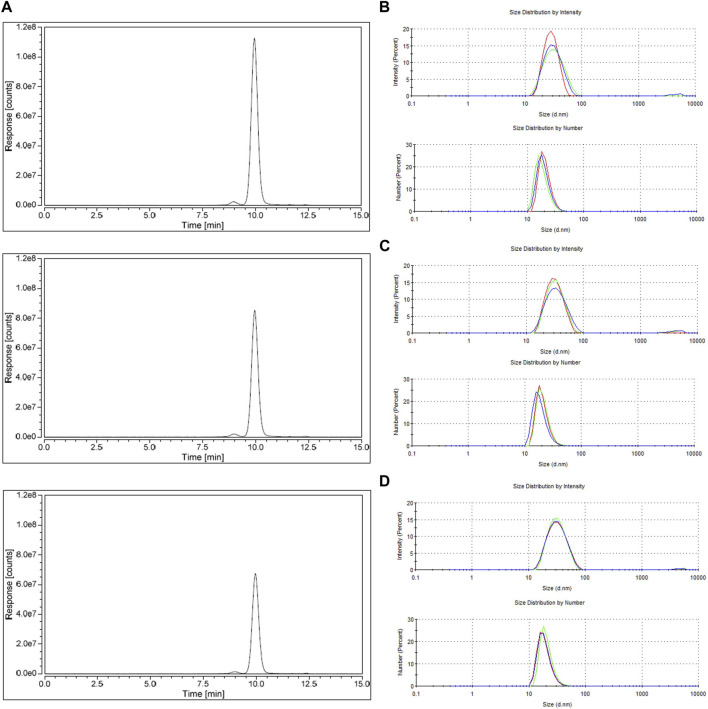
Characterization of the eluted peaks from reproducibility study (residence time of 4.8 s, loading up to DBC 10%) by: **(A)** size exclusion chromatography (top, run 1; middle, run 2; bottom, run 3) **(B–D)** size distributions of eluate run 1, eluate run 2, and eluate run 3, respectively, (top, distribution by intensity; and bottom, distribution by number).

The eluates were further characterized by SDS-PAGE and western blot to assess purity and identity. Additionally, transmission electron microscopy (TEM), HPLC-SEC and DLS were also performed to assess capsid morphology, the presence of aggregates, particle size and size distribution. The results are presented in Figures 5, 6; Table 2. Finally, the reduction in total protein and ds-DNA was analysed during the chromatographic step (Table 1).

**TABLE 2:** Relative area of HPLC-SEC peaks from reproducibility experiments.

Run	Relative area (%)	
	Peak 1	Peak 2
1	1.99	98.01
2	2.20	97.80
3	2.03	97.97

The main AAVs capsids proteins, VP1, VP2, and VP3 are present in SDS-PAGE (Figure 5A), for each eluate and its identity is confirmed by western blot (Figure 5B), with the expected proportion of 1:1:10 (VP1:VP2:VP3). No visible impurities are seen in the SDS-PAGE. TEM images (Figure 5C) show that the size, shape, and capsid integrity of AAVs particles are kept between runs. The analysis of size exclusion chromatography (Figure 6A; Table 1) reveals two peaks. The first one (smaller) with a relative area of 2% at 9.0 min is indicative of the presence of aggregates, although this is not visible in TEM analysis. The second peak, with a retention time of 12.0 min and a relative area of 98%, corresponds to the AAVs purified. The observable difference in peak height of the HPLC-SEC chromatograms (Figure 6A) is explained by the different volumes of buffer added to dilute and neutralize the elution pools. This dilution is also noticeable in the TEM analysis (Figure 5). Regarding the characterization by particle size distribution (Figures 6B–D), the eluted AAVs have an average diameter of 26 nm. The differences observed in the peak shape, between intensity and number distributions, are due to the presence of large particles (aggregates) above 1 μm, that cause a peak extension, confirming the SEC results. Regarding the removal of total protein and ds-DNA (Table 1), fibro affinity units allowed a substantial reduction of 78–79% of total protein and 80–90% of ds-DNA.

The nanofiber adsorbents evaluated for AAV5 affinity capture showed that it is possible to operate at flow rates as high as 15 mL min^−1^ (RT = 1.6 s), with pressure drops lower than 0.4 MPa. On one hand, these values are in accordance with those reported for affinity purification of monoclonal antibodies with similar cellulose-based nanofiber adsorbents (Cytiva, 2020b). On the other hand, the nanofiber adsorbents for AAVs allow 10 times higher operational flow rates in comparison to packed beds of resin with 2.5 times its volume (Cytiva, 2018).

The nanofiber adsorbents reveal a good dynamic binding capacity and recovery yields even at low residence times in comparison to packed beds. Mendes et al., using periodic counter-current chromatography, reported an AAVs recovery of 82.8% using Capto AVB™ of 0.2 mL, with 10-fold higher residence times in comparison to the used nanofibers (Mendes et al., 2022). With respect to the experimental binding capacity, the highest value (6.08 × 10^14^ TP mL^−1^) was obtained for a residence time of 4.8 s. Although capacity is in the same order of magnitude as what is reported by some manufacturers there is a substantial improvement in diminished residence times Cytiva, 2018; Cytiva, 2020a; Fisher Scientific, 2017; Repligen Corporation, 2022). To the present moment there are no reports of convective materials functionalized with affinity ligands for AAVs purification. However, a great effort has been made in the development of new ligands for different AAVs serotypes (Zhao et al., 2019; Chevrel et al., 2022). Thus, the cellulose-based nanofibers adsorbents reported here, arise as the first convective adsorber for AAVs affinity purification, enabling a meaningful removal of impurities even processing a large volume of AAVs-clarified material with a single chromatography step. To enable the application of the nanofiber advantages to the larger-scale of AAVs production, the scale-up of the device of 0.4 mL is under development using the same principles of HiTrap Fibro™ PrismA (Cytiva, 2020b).

## 4 Conclusion

In this work, the affinity purification of AAV5 clarified material using nanofiber adsorbents was evaluated in this work. The mathematical modelling of the breakthrough curves reveals that the dynamic binding capacity of nanofiber adsorbents can range from 1.0 × 10^14^ TP mL^−1^ (at the lowest RT scoped) up to 6.08 × 10^14^ TP mL^−1^ (at the highest RT scoped), approaching the saturation capacity of 8.29 × 10^14^ TP mL^−1^ for RT above 30 s. These nanofiber adsorbents enable a 10-fold reduction in residence time in comparison to packed-bed chromatography, with similar AAVs recoveries and impurity removals. All in all, the nanofiber adsorbents studied enable the possibility of processing large volumes of clarified products in less time (large volume reduction). This can contribute to eliminating the need for a previous concentration step before the affinity capture, usually performed by tangential flow filtration. This simplification of the purification process reduces buffer consumption and the need for storage tanks and hold points, therefore increasing the global process yield and reducing processing times and costs.

## Data Availability

The original contributions presented in the study are included in the article/Supplementary Material, further inquiries can be directed to the corresponding author.
