# Supplementary material for: Efficient adeno-associated virus serotype 5 capture with affinity functionalized nanofiber adsorbents
Source: Front Bioeng Biotechnol. 2023 May 16;11:1183974. doi: 10.3389/fbioe.2023.1183974 (PMC10229133; doi:10.3389/fbioe.2023.1183974)
Supplement: Supplementary file 1 [file DataSheet1.pdf]

Supplementary Material

Efficient adeno-associated virus serotype 5 capture with affinity functionalized nanofiber adsorbents

Salomé Neto[1,2], João P. Mendes[1,2], Susana B dos Santos[3], Anita Solbrand[4], Manuel J. T. Carrondo[1], Cristina Peixoto[1,2], Ricardo J.S. Silva[1,2*]

[1]iBET, Instituto de Biologia Experimental e Tecnológica, Oeiras, Portugal

[2]ITQB NOVA, Instituto de Tecnologia Química e Biológica António Xavier, Universidade Nova de Lisboa, Av. da República, 2780-157 Oeiras, Portugal

[3]Cytiva, UK

[4]Cytiva, 751 84 Uppsala, Sweden

*** Correspondence:**
Ricardo J.S. Silva
rsilva@ibet.pt

1 Supplementary Figures and Tables

1.1 Supplementary Figures

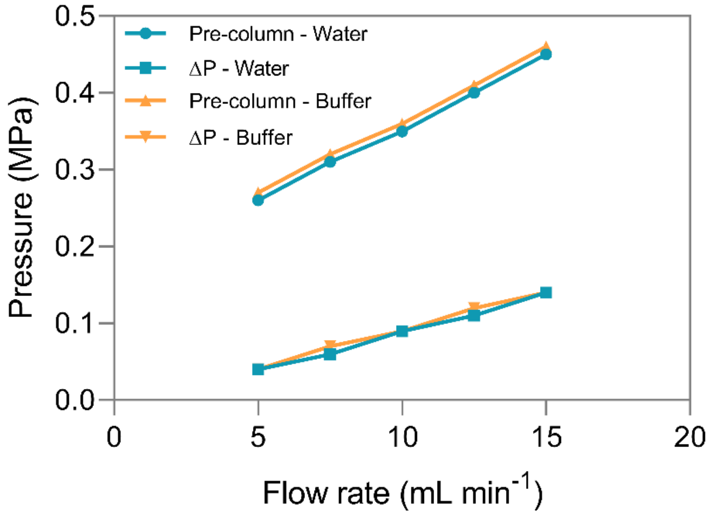

Supplementary Figure 1. Pre-column and ΔP pressures for nanofiber adsorbents with water and equilibrium buffer at flow rates 5.0, 7.5, 10.0, 12.5 and 15.0 mL min^{-1}.

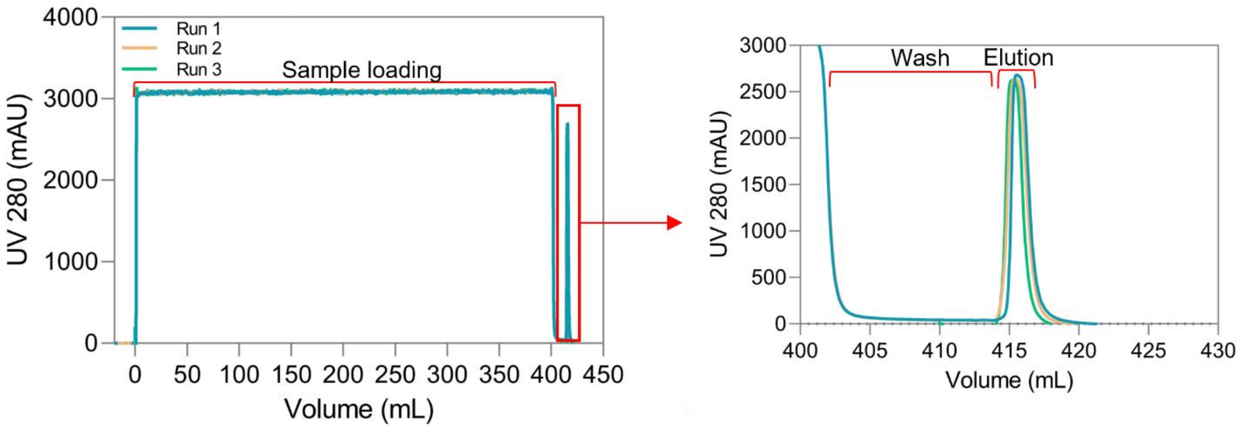

Supplementary Figure 2. UV absorption at 280 nm of three runs from reproducibility experiment, at a residence time of 4.8 seconds and with a loading volume corresponding to $DBC_{10\%}$ (400 mL or 1000 CV).
